# Association of Serum Adiponectin with Intima Media Thickness of Dorsalis Pedis Artery and Macroangiopathy in Type 2 Diabetes

**DOI:** 10.1155/2020/4739271

**Published:** 2020-01-03

**Authors:** Tao Chen, Mei Tu, Lihua Huang, Youping Zheng

**Affiliations:** ^1^Department of Endocrinology and Metabolism, Affiliated Longyan First Hospital of Fujian Medical University, Longyan, 364000 Fujian Province, China; ^2^Department of Tumor Radiotherapy, Affiliated Longyan First Hospital of Fujian Medical University, Longyan, 364000 Fujian Province, China; ^3^Department of Ultrasound, Affiliated Longyan First Hospital of Fujian Medical University, Longyan, 364000 Fujian Province, China

## Abstract

**Objective:**

To investigate the association of the serum adiponectin level with the intima media thickness of the dorsalis pedis artery (D-IMT) and macroangiopathy in type 2 diabetes (T2DM).

**Methods:**

We recruited 173 patients with T2DM, of whom 83 had macroangiopathy (MA group) and 90 did not have macroangiopathy (NM group), and 40 normal control subjects (NC group). We measured D-IMT using color B-mode Doppler ultrasonography. Serum adiponectin, blood glucose, lipids, and other clinical characteristics were analyzed. Participants were divided into three subgroups according to serum adiponectin level (high, moderate, and low).

**Results:**

Compared with the NM and NC groups, serum adiponectin levels were significantly decreased in the MA group after adjusting for sex and body mass index. Compared with the NM and NC groups, D-IMT was significantly increased in the MA group. Compared with the moderate- and high-adiponectin subgroups, D-IMT was significantly increased in the low-adiponectin subgroup. The prevalence of diabetic macroangiopathy increased gradually with decreasing adiponectin levels. After controlling for age, sex, smoking, and alcohol drinking, partial correlation analysis showed that adiponectin was negatively correlated with D-IMT. Elevated serum adiponectin was independently associated with a decreased risk for diabetic macroangiopathy by logistic regression analysis. Multiple linear regression analysis revealed that adiponectin was an independent factor of D-IMT. In receiver operating characteristic analyses, the area under the curve for traditional risk factors plus adiponectin for prediction of macroangiopathy was 0.984, while that of traditional risk factors alone was 0.972.

**Conclusions:**

Adiponectin is lower in patients with T2DM with macroangiopathy. We suggest that D-IMT could represent a noninvasive indicator of diabetic macroangiopathy. Decrease of adiponectin as an independent risk factor for both macroangiopathy and D-IMT among Chinese patients with T2DM suggests that adiponectin might have clinical utility in the prediction of diabetic macroangiopathy. This clinical trial is registered in the “Chinese Clinical Trial Registry.” The registration number is ChiCTR-ROC-17011731.

## 1. Introduction

Type 2 diabetes mellitus (T2DM) has a serious impact on human health and has become a global public health problem. Diabetic macroangiopathy is the primary complication contributing to death in patients with T2DM, accounting for 60–70% of deaths among patients with T2DM [1–3]. Compared with type 1 diabetes, the prevalence of diabetic macroangiopathy is higher and affected by more factors in the early phases of T2DM. Atherosclerosis (AS) is well known to represent the pathological basis of macrovascular complications and mainly involves the aorta and medium-sized arteries such as the coronary, cerebral, renal, and lower-extremity arteries. Thus, AS can lead to coronary heart disease, cerebrovascular accident, lower-extremity arteriosclerosis, occlusive disease, and so on. Intima media thickness (IMT) can be measured as a noninvasive index to evaluate early AS and also provides an effective predictor of cardiovascular events [4–6]. It has been reported that AS begins in arteries below the knee, such as the dorsalis pedis and anterior tibial arteries [7]. Lower-extremity vascular disease has been observed in the early stages of diabetes, which will increase the mortality associated with T2DM [8]. The dorsalis pedis artery is the most frequent site of macrovascular lesions in patients with diabetes [9], and the rate of dorsalis pedis artery involvement in diabetic macroangiopathy has been reported to be as high as 63.4% [10]. The pulse of the dorsalis pedis artery can reflect the risk of macrovascular disease [11].

Most previous studies have measured the IMT of the carotid artery; studies reporting measurements of the IMT to evaluate diabetic macrovascular complications of the lower-extremity vasculature are scarce. Specifically, few studies have focused on the dorsalis pedis artery in diabetic macroangiopathy, and IMT data of this artery is particularly lacking in patients with AS. Therefore, this study is aimed at measuring the IMT of the dorsalis pedis artery.

Adiponectin is an endogenous, biologically active protein produced by adipose, which represents 0.01% of the total cell protein content [12]. Serum concentrations may be 1.9–17 mg/L [13], and adiponectin has been found to have antiatherosclerotic, anti-inflammatory, and lipid regulatory properties. Thus, adiponectin contributes to protecting endothelial cells and improving insulin resistance [14, 15].

There is very little information about the role of serum adiponectin and the IMT of the dorsalis pedis artery (D-IMT) in the context of diabetic macroangiopathy. In the present study, we compared the effects of adiponectin and other relevant factors on macrovascular complications. We also investigated the associations of serum adiponectin with D-IMT and macroangiopathy in T2DM and evaluated the predictive value of decreased serum adiponectin for diabetic macroangiopathy.

## 2. Material and Methods

### 2.1. Ethics Statement

This study protocol was approved by the institutional review board of the Affiliated Longyan First Hospital of Fujian Medical University. Written informed consent was obtained from each participant before the study.

### 2.2. Study Population

We recruited a total of 213 people for this cross-sectional, observational study between June 2017 and October 2018 from the Affiliated Longyan First Hospital of Fujian Medical University. All participants were Chinese. The cohort consisted of 173 individuals with T2DM who were recruited from the outpatient clinic or inpatient ward of the Department of Endocrinology and Metabolism. Inclusion criteria were age over 20 years, known T2DM with lifestyle modification or newly diagnosed T2DM according to the World Health Organization criteria (1999), and/or undergoing treatment with insulin or oral hypoglycemic agents [16]. Patients with T2DM were allocated to a macroangiopathy (MA) group (*n* = 83) or a nonmacroangiopathy (NM) group (*n* = 90). Exclusion criteria for the MA group were as follows: acute infectious disease such as cold or diarrhea, uncontrolled hypertension, nonhomeostasis such as diabetic ketoacidosis, hyperosmolar syndrome or hypoglycemia, severe dyslipidemia (total cholesterol (TC) > 10.34 mmol/L, triglyceride (TG) level > 5.65 mmol/L), clear microvascular lesions such as diabetic nephropathy or retinopathy, autoimmune disease or malignant tumor, or any medical condition requiring immediate and active treatment. Participants with T2DM were assigned to the MA group if at least one of the following criteria were met: diagnosis of coronary heart disease, cerebral infarction, or peripheral vascular ischemic disease by the attending medical specialist according to clinical symptoms, signs, and auxiliary examination; cerebral infarction lesions identified by brain magnetic resonance imaging; atherosclerotic plaques of the peripheral blood vessels (bilateral carotid artery, lower-extremity artery, etc.) identified by color Doppler ultrasound examination; and ischemic myocardial injury detected by electrocardiogram (ECG), dynamic electrocardiogram, echocardiography, or percutaneous coronary angiography.

Forty healthy individuals were recruited from the Health Examination Center as the normal control (NC) group. Exclusion criteria for the NC group were DM, coronary heart disease, cerebral infarction, hypertension, peripheral vascular disease, other major organic diseases of the liver and kidney, and tumor. Examination using B-mode ultrasound was carried out to assess the presence of atherosclerotic plaques in the lower extremity and carotid arteries in the NC group.

### 2.3. Assessment of Anthropometric and Clinical Characteristics and Laboratory Measurements

Detailed medical history was recorded and a physical examination conducted for all participants. Duration of diabetes was calculated as the time the first date of definitive diagnosis with T2DM to the date of enrollment. Cigarette smoking was defined as having smoked at least 100 cigarettes in one's lifetime. Alcohol drinking was defined as consumption of at least 30 g of alcohol per week for 1 year or more. Body weight and height were measured to the nearest 0.1 kg and 0.01 m, and the body mass index (BMI) was calculated as body weight (kg) divided by height squared (m^2^). Systolic blood pressure (SBP) and diastolic blood pressure (DBP) were measured after the participant had rested in a sitting position for at least 30 minutes. Blood samples were collected in the morning after overnight fasting for 8–10 hours. Fasting plasma glucose (FPG), postprandial 2 hours blood glucose (PPG), TC, TG, high- and low-density lipoprotein cholesterol (HDL-C and LDL-C), and uric acid (UA) were routinely tested using an autoanalyzer (Hitachi 7600-100, Japan). Glycosylated hemoglobin (HbA_1_C) was measured using high-performance liquid chromatography (G8 HPLC Analyzer; Tosoh, Tokyo, Japan). Fibrinogen (Fg) and high-sensitivity C-reactive protein (hs-CRP) were measured using a particle-enhanced immunoturbidimetric assay (Hitachi High-Technologies Corp., Tokyo, Japan). Fasting insulin (Fins) was measured by immunochemical detection with electroluminescence (Olympus AU2700, Tokyo, Japan). The degree of insulin resistance was assessed using the homeostasis model assessment 2 of insulin resistance (HOMA2-IR), which was calculated using computer software after inputting Fins and FPG data. The software can be downloaded from the Oxford Center of Diabetes, Endocrinology and Metabolism [17].

### 2.4. Measurement of Serum Adiponectin

Blood samples (2 mL) were collected after fasting from the elbow vein and placed in a static status for 30 minutes at room temperature, then stored at −80°C after centrifuging for 10 min at 4000 rpm in Eppendorf tubes. Repeated freezing and thawing were avoided. In order to reduce measurement error, serum adiponectin levels were analyzed in one session when all samples were collected. Measurement was carried out using a commercially available enzyme-linked immunosorbent assay (ELISA) kit (ALPCO Diagnostics, Salem, NH, USA). Intra- and interassay coefficients of variation (CVs) for the kit were 4.8 and 5.2%, respectively. Following measurement, participants were divided into three subgroups according to serum adiponectin level: high-adiponectin subgroup (>3.27 mg/L), moderate-adiponectin subgroup (1.85–3.27 mg/L), and low-adiponectin subgroup (<1.85 mg/L).

### 2.5. Ultrasound Measurements of Intima Media Thickness of the Dorsalis Pedis Artery

We measured D-IMT using a LOGIQ 9 ultrasonograph (GE Healthcare, Wauwatosa, WI, USA) equipped with a 7.5 MHz linear transducer, set to the condition of lower-limb artery and single-point-aggregation mode. Participants were required to maintain adequate exposure of the examination site in the supine position so that the pulse of the dorsalis pedis artery could be felt 2–3.5 cm below the connection of the internal and external ankle joint. Measurement of D-IMT was performed at the site of greatest thickness and at two other points, 1 cm upstream and downstream of this site. Therefore, six sites were assessed in the left and right dorsalis pedis arteries for each participant. The final measurement of D-IMT was defined as the mean value of the above six sites. Measurements of D-IMT of all participants were recorded by the same sonographer, and another senior expert was invited to check the database. These two sonographers were blinded to participants' clinical characteristics.

### 2.6. Statistical Analysis

All the results were analyzed using the SPSS statistical package, version 19.0 (SPSS Inc., Chicago, IL, USA). Continuous data which exhibited normal distribution are presented as mean ± standard deviation (SD). Median (interquartile ranges) is used to present nonnormally distributed continuous variables. Categorical variables are presented as number (%). Clinical characteristics of the groups were compared using analysis of variance for continuous and normally distributed variables and the *χ*^2^ test for categorical variables. The Kruskal-Wallis rank sum test was used to analyze variables with abnormal distribution. Partial correlation analysis was used to compare the strength of associations between risk factors and serum adiponectin level after adjusting for sex, age, cigarette smoking, and alcohol drinking. Logistic and linear regression models were used to test the associations of adiponectin with diabetic macroangiopathy and D-IMT, respectively. Three multivariable models were tested: In Model 1, age, sex, BMI, cigarette smoking, HbA_1_C, SBP, TG, HDL-C, and LDL-C were included. In Model 2, adiponectin was included. In Model 3, age, sex, BMI, cigarette smoking, HbA_1_C, SBP, TG, HDL-C, LDL-C, and adiponectin were included. Receiver operating characteristic (ROC) curves of the above models were generated to evaluate the predictive value of relative risk factors for diabetic macrovascular complications. Statistical significance was accepted for two-tailed *P* values of <0.05.

## 3. Results

### 3.1. Demographic Characteristics of All the Participants

There was no significant difference in terms of sex, age, cigarette smoking, or alcohol drinking between the three groups (Table 1).

### 3.2. Comparison of General Clinical Data and Intima Media Thickness of the Dorsalis Pedis Artery

Examination using B-mode ultrasound revealed no atherosclerotic plaques among the NC group. The MA and NM groups were found to have higher mean BMI, HbA_1_C, TC, and lower mean HDL-C than the NC group, but there was no significant difference in the above variables between the MA and NM groups. Diabetes duration was higher in the MA than the NM group. The MA and NM groups exhibited significantly increased FPG, PPG, SBP, DBP, UA, Fins, HOMA2-IR, Fg, hs-CRP, and D-IMT compared with the NC group, and the above variables were also significantly higher in the MA group than the NM group. The mean TG was significantly higher in the MA group than the NC group, and LDL-C was significantly increased in the MA group compared with both the NC and NM groups (Table 1).

### 3.3. Comparison of Serum Adiponectin

Compared with the NM and NC groups, serum adiponectin was significantly decreased in the MA group. Meanwhile, the adiponectin level was also significantly lower in the NM group than the NC group. The results were similar even after stratification according to sex or BMI. After sex stratification, adiponectin levels were found to be higher in women than men among the MA group, but no significant differences were observed between men and women of the NM or NC group. Guidelines for the prevention and control of overweight and obesity in Chinese adults were used to stratify participants by BMI; the guidelines define BMI ≥ 24 kg/m^2^ as overweight or obese and BMI < 24 kg/m^2^ as normal [18]. After BMI stratification, we found that the serum adiponectin levels of overweight and obese participants were lower than those of normal participants in every group (Table 2).

### 3.4. Comparison of Demographic Characteristics among the New Subgroups

There were no significant differences in terms of sex, age, cigarette smoking, and alcohol drinking between the three subgroups (Table 3).

### 3.5. Comparison of Biochemical Indexes and Intima Media Thickness of the Dorsalis Pedis Artery of the Three Subgroups

There were no significant differences in term of Fg among the three subgroups. BMI, DBP, FPG, PPG, HbA_1_C, TG, and SBP were significantly lower, but HDL-C was significantly higher in the high-adiponectin subgroup than the moderate- and low-adiponectin subgroups. The above indexes were not significantly different between the moderate- and low-adiponectin subgroups.

We observed significantly higher D-IMT in the low-adiponectin subgroup than in the moderate- or high-adiponectin subgroups, and the moderate-adiponectin subgroup also exhibited significantly higher D-IMT than the high-adiponectin subgroup. Furthermore, the prevalence of diabetic macroangiopathy increased gradually as the level of adiponectin decreased in the three subgroups. The incidence of macrovascular complications increases from the -high- to -low-adiponectin subgroups (*χ*^2^ = 45.522, df = 2, *P* < 0.001) (Table 3).

### 3.6. Partial Correlation Analysis

Partial correlation analysis revealed that serum adiponectin was negatively correlated with D-IMT and positively correlated with HDL-C (Table 4).

### 3.7. Logistic Regression Analysis for Diabetic Macroangiopathy

Among the traditional risk factors, our study confirmed a significant correlation between SBP and the risk of diabetic macroangiopathy. In logistic regression analysis, elevated adiponectin levels were significantly associated with a risk of diabetic macroangiopathy (odds ratio (OR) 0.474, 95% confidence interval (CI) 0.346–0.648). We further adjusted the risk factors in the model and found that the increased serum adiponectin was still significantly correlated with the risk of diabetic macroangiopathy (OR 0.293, 95% CI 0.113–0.759), suggesting that it was an independent risk factor for macroangiopathy. In addition, sex and SBP also showed correlation with macrovascular disease (Table 5).

### 3.8. Multivariate Linear Regression Analysis for Intima Media Thickness of the Dorsalis Pedis Artery

Linear regression indicated a significant correlation between SBP and D-IMT in the traditional factors model. The linear regression model with adiponectin alone suggested that adiponectin was significantly correlated with D-IMT. After adjustment for traditional factors, adiponectin was still an independent influencing factor for D-IMT (Table 6).

### 3.9. Receiver Operating Characteristic Curve Analysis

**Figure 1:**
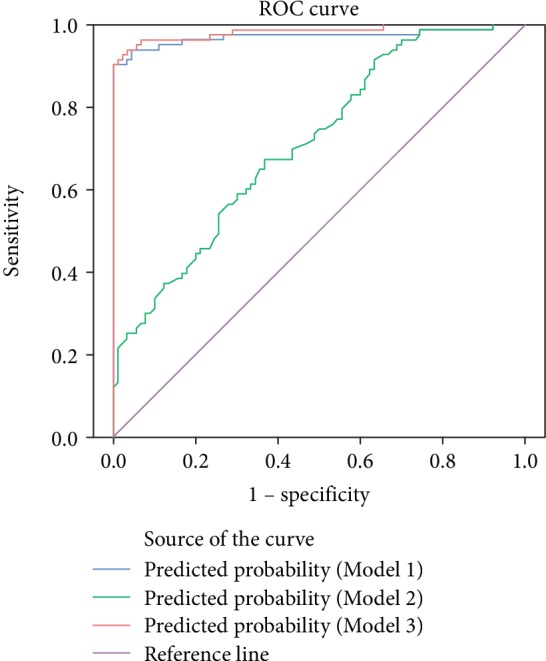
Receiver operating characteristic curve analysis for macroangiopathy prediction with three different models. Model 1 included traditional macrovascular risk factors such as age, sex, cigarette smoking, body mass index (BMI), HbA_1_C, systolic blood pressure (SBP), triglycerides (TG), high-density lipoprotein cholesterol (HDL-C), and low-density lipoprotein cholesterol (LDL-C). Area under the curve (AUC) of Model 1: 0.972, 95% CI: 0.943~1.000, *P* < 0.001. Model 2 included only adiponectin. AUC of Model 2: 0.714, 95% CI: 0.639~0.789, *P* < 0.001. Model 3 incorporated Model 1 plus Model 2. AUC of Model 3: 0.984, 95% CI: 0.965~1.000, *P* < 0.001.

Areas under ROC curves of the model including traditional risk factors only, the model including adiponectin only, and the model including traditional risk factors and adiponectin were 0.972, 0.714, and 0.984, respectively (Figure 1).

## 4. Discussion

According to the latest epidemiological survey, individuals with diabetes account for 9.7% of the total population of China and those with prediabetes account for 15.5% [19]. Macrovascular and microvascular complications are the leading causes of death in T2DM. Results of the Diabetes Control and Complications Trials (DCCT) and the UK Prospective Diabetes Study (UKPDS) have shown that simply controlling blood glucose can reduce microvascular complications of diabetes, although this may not significantly reduce the risk of macrovascular complications of T2DM [20]. Therefore, it is important to prevent and control macrovascular complications in T2DM. Compared with nondiabetic patients, lower-extremity arterial disease is more distal in patients with diabetes and is associated with higher rates of amputation and death [21]. The dorsalis pedis artery is the first artery to exhibit diabetic macrovascular lesions and has a higher incidence of lesions than popliteal or anterior/posterior tibial arteries [7, 10]. Increased IMT is an early pathological change in the development of macrovascular lesions [22]. In this study, D-IMT was significantly higher in the MA group compared with the NM and NC groups and higher in the NM group than the NC group. Our findings support the results of Gan et al., who reported that D-IMT was thicker in diabetic patients than nondiabetic individuals [23]. Furthermore, we found that the D-IMT value was significantly increased in diabetic patients with macroangiopathy compared with those without. Therefore, the dorsalis pedis artery can be considered to be representative of the whole-body vasculature in T2DM, which has significance for early screening.

Adiponectin receptors (AdipoR1 and AdipoR2) can combine with adiponectin to activate the p38 mitogen-activated protein kinase (p38MAPK) pathway, thus playing a physiological and multifunctional role after activation of the peroxisome proliferator activated receptors *α* (PPAR-*α*). Adiponectin can be considered to be a key factor in the association between metabolic and macrovascular diseases [24] and is known to function as an anti-inflammatory in vascular endothelial cells, increase insulin sensitivity, have antiatherosclerotic properties, reverse myocardial remodeling, and so on [25].

Serum adiponectin levels were lowest in patients with T2DM with macrovascular lesions in our study, and patients with T2DM but without diabetic macroangiopathy presented lower adiponectin levels than nondiabetic controls. After stratifying by sex or BMI, adiponectin level remained lower in patients with diabetic macroangiopathy than in those without macrovascular lesions. Further study revealed that the prevalence of macrovascular disease was up to 64.79% among the participants in the low-adiponectin subgroup, which was significantly higher than in moderate- and high-adiponectin subgroups. The prevalence of macrovascular disease was also significantly higher in the moderate-adiponectin subgroup than in the high-adiponectin subgroup. Furthermore, logistic regression analyses revealed that adiponectin is an influencing factor for type 2 diabetic macroangiopathy. This is consistent with the latest research findings. Adiponectin levels are known to be significantly lower in patients with macrovascular lesions than in healthy individuals. Hypoadiponectinemia is a significant risk factor in the progress of macrovascular disease and is also an independent predictor of lesion size, extent of disease, and prognosis [26, 27]. Studies have shown that adiponectin plays a role in preventing the development of macrovascular diseases [28], and ROC analysis in the present study found the area under the curve of traditional risk factors such as blood glucose, blood pressure, blood lipids, and cigarette smoking to increase after adiponectin was added to the risk factors, suggesting that adiponectin level is a predictor of diabetic macroangiopathy.

Our study found that D-IMT was significantly higher in the low-adiponectin subgroup than the moderate- or high-adiponectin subgroups. Multiple linear regression analysis confirmed adiponectin to be an independent risk factor for D-IMT, showing negative correlation. Matsuda et al. studied transgenic mice with an adiponectin defect (adipo[-/-]) and found that the formation of new vascular intima was twice that of wild-type mice after vascular injury [29]. Immunohistochemical studies conducted by Okamoto et al. showed that adiponectin carried by adenovirus was transferred into the foam cells of atherosclerotic lipid streaks, inhibiting the formation of atherosclerotic plaques [30]. Studies have shown that tumor necrosis factor- (TNF-) *α* secretion from human aortic endothelial cells is inhibited by adiponectin in a dose-dependent manner. Further studies have shown that adiponectin causes increased accumulation of cyclic adenosine monophosphate-protein kinase A (cAMP-PKA) in endothelial cells, inhibiting rapid phosphorylation and degradation of TNF-*α*-mediated nuclear transcription factor (NF-*κ*B) suppressor molecules (I*κ*B-*α*), thus reducing activation of NF-*κ*B [31]. A 5-year prospective study showed that hypoadiponectinemia was an independent predictor of progression of IMT [32]. Adiponectin may exert its antiatherosclerotic actions by reducing oxidative stress, repairing endothelial injury, acting as an endogenous anti-inflammatory mediator to regulate acquired immunity, and inducing macrophages and T cells to produce anti-inflammatory effects [33–36].

Although adiponectin is secreted by adipose tissue, secretion is reduced in obese patients. This study reveals that levels of adiponectin are significantly lower in overweight and obese patients than in people with normal BMI after stratification by BMI in each group. This is in agreement with a previous study [37]. Partial correlation analysis suggested a negative correlation between BMI and adiponectin, therefore implying that adiponectin levels can be effectively improved by lifestyle modifications such as weight reduction and calorie control, especially for obese patients with T2DM [38].

A previous study has reported that adiponectin levels are influenced by sex and that adiponectin levels are significantly lower among males than females. Our study found that sex differences in terms of adiponectin level existed only in the diabetic macroangiopathy group; no statistically significant differences were found in the other two groups. The reason for this is unknown; it may be that the level of adiponectin is affected by many factors and that sex is not the most important of all the influencing factors. Other reasons such as race and sample-selection bias may have influenced the result. Further studies are required in order to confirm whether adiponectin is sex-dependent in Chinese people. The reason for the sex difference of adiponectin has not been clarified. Nishizawa et al. proposed that high levels of androgenic hormone in males might lead to the result [39], but a recent study found that sex differences still existed even when men were compared with postmenopausal women [40]. This makes the effect of sex hormone differences between men and women on adiponectin levels questionable.

The present study found the incidence of diabetic macroangiopathy to be lower in women than men, which is consistent with many previous studies. The Framingham study found that macrovascular morbidity and mortality rates in women lagged behind men by 10 years and revealed that the incidence of cardiovascular disease increases rapidly after menopause, suggesting a protective effect of estrogen [41]. Female estrogen has a protective effect on large blood vessels as it promotes the release of nitric oxide and consequent vasodilation, regulating the production of prostaglandin and inhibiting the proliferation of smooth muscle [42]. We noted sex differences in macroangiopathy as well as adiponectin levels and thus hypothesized that the differences in diabetic macroangiopathy might be achieved through the adiponectin pathway. Further research is needed to confirm this speculation.

Hypertension was found to be an independent risk factor for diabetic macroangiopathy in this study, consistent with the results of Fariba et al. [43]. The Action in Diabetes and Vascular Disease: Preterax and Diamicron Controlled Evaluation (ADVANCE) trial also showed that aggressive antihypertensive treatment significantly reduced the incidence of the primary endpoint by 9% in patients with diabetes, and the risk of macrovascular disease was reduced by 8% [44]. Hypertension and diabetes have synergistic effects, which can aggravate vascular intima injury, promote the thickening of IMT, and accelerate the formation of atherosclerosis [45].

In the present study, we found that hs-CRP levels of patients with T2DM with macroangiopathy were higher than those without. The hs-CRP level was higher in the low-adiponectin subgroup than the moderate- and high-adiponectin subgroups. Partial correlation analysis suggested a negative correlation between hs-CRP and adiponectin. Thus, our results support our hypothesis that T2DM and AS both exist in chronic low-grade inflammation [46, 47]. The hs-CRP level represents a nonspecific, sensitive index of inflammation, which is increased in the context of AS. Elevated serum hs-CRP levels have even been shown to predict the occurrence of cardiovascular events in previous studies [48].

There are some limitations in our research which should be acknowledged. First, because of the observational nature of this study, it was not easy to assess the direct, causative role of adiponectin. Second, some personal lifestyle factors such as physical activity and calorie intake, which can influence adiponectin levels, were not taken into consideration. Third, in this study, we adjusted for sex and BMI. Adiponectin is a cytokine secreted by adipose tissue; therefore, the instrument used to measure body fat can be used to quantify adipose tissue, which would be a better research design to adjust for obesity-relevant factors. Fourth, this study was conducted at a single center for diabetic complications. More in-depth and long-term follow-up of our cohort is needed to reveal the effects of adiponectin and relevant risk factors on diabetic macrovascular complications.

## 5. Conclusions

In brief, adiponectin may play a key role in preventing and treating diabetic macroangiopathy by reducing the inflammatory mediators and repairing the vascular endothelium thus inhibiting AS. Therefore, we suggest that patients with T2DM should be monitored for D-IMT as a noninvasive objective indicator, to facilitate early detection of subclinical macroangiopathy. Adiponectin may have clinical utility as an effective predictor of diabetic macroangiopathy in the future.

## Figures and Tables

**Table 1:** Comparison of clinical characteristics among different groups.

Clinical measurement	Normal control (NC group, *n* = 40)	Diabetes without macroangiopathy (NM group, *n* = 90)	Diabetes with macroangiopathy (MA group, *n* = 83)	*P* value
Age (y)	50.98 ± 13.83	53.81 ± 11.87	54.49 ± 8.37	0.247
Male, *n* (%)	18 (45.0)	44 (48.9)	38 (45.8)	0.886
Cigarette smoking (%)	13 (32.5)	34 (37.8)	33 (39.8)	0.737
Alcohol drinking (%)	25 (62.5)	60 (66.7)	55 (66.3)	0.891
BMI (kg/m^2^)	22.04 ± 2.95	24.31 ± 2.85^∗^	24.61 ± 3.23^∗^	<0.001
Diabetes duration (y)	—	5 (1, 8.5)	8 (4.33, 14)^#^	<0.001
SBP (mmHg)	117.25 ± 7.90	130.60 ± 4.39^∗^	159.70 ± 14.23^∗#^	<0.001
DBP (mmHg)	74.70 ± 6.53	81.99 ± 4.09^∗^	90.24 ± 9.48^∗#^	<0.001
FPG (mmol/L)	5.33 ± 0.56	9.13 ± 2.45^∗^	10.20 ± 2.74^∗#^	<0.001
PPG (mmol/L)	6.34 ± 0.67	14.49 ± 3.63^∗^	15.85 ± 4.08^∗#^	<0.001
HbA_1_C (%)	5.50 ± 0.63	8.67 ± 1.61^∗^	8.83 ± 1.73^∗^	<0.001
TC (mmol/L)	4.36 ± 0.73	4.94 ± 1.13^∗^	5.13 ± 0.94^∗^	<0.001
TG (mmol/L)	1.31 ± 0.78	1.57 ± 0.76	1.74 ± 0.99^∗^	0.036
HDL-C (mmol/L)	1.32 ± 0.39	1.14 ± 0.26^∗^	1.09 ± 0.29^∗^	<0.001
LDL-C (mmol/L)	2.93 ± 0.87	3.14 ± 0.88	3.44 ± 0.92^∗#^	0.008
UA (mmol/L)	309.68 ± 92.27	343.85 ± 73.26^∗^	386.96 ± 90.07^∗#^	<0.001
Fg (g/L)	3.06 ± 0.94	3.53 ± 0.87^∗^	4.02 ± 1.02^∗#^	<0.001
Fins (*μ*U/mL)	35.73 ± 7.83	72.90 ± 14.89^∗^	155.16 ± 41.30^∗#^	<0.001
HOMA2-IR	0.67 ± 0.14	1.53 ± 0.31^∗^	3.33 ± 0.78^∗#^	<0.001
hs-CRP (mg/L)	0.92 (0.74, 2.14)	2.14 (1.00, 3.83)^∗^	4.02 (2.14, 5.75)^∗#^	<0.001
D-IMT (cm)	0.0276 ± 0.0099	0.0538 ± 0.0075^∗^	0.1135 ± 0.0222^∗#^	<0.001

^∗^Significantly different from the NC group. ^#^Significantly different from the NM group. BMI: body mass index; SBP: systolic blood pressure; DBP: diastolic blood pressure; FPG: fasting plasma glucose; PPG: postprandial 2 hours blood glucose; HbA_1_C: glycosylated hemoglobin; TC: total cholesterol; TG: triglyceride; HDL-C: high-density lipoprotein cholesterol; LDL-C: low-density lipoprotein cholesterol; UA: uric acid; Fg: fibrinogen; Fins: fasting insulin; HOMA2-IR: homeostasis model assessment 2 of insulin resistance; hs-CRP: high-sensitivity C-reactive protein; D-IMT: intima media thickness of the dorsalis pedis artery. Measurement data are presented as mean ± standard deviation or median (with interquartile ranges).

**Table 2:** Comparison of serum adiponectin levels after sex and BMI stratification.

Cohort	Normal control (NC group, *n* = 40)	Diabetes without macroangiopathy (NM group, *n* = 90)	Diabetes with macroangiopathy (MA group, *n* = 83)	*P* value
Overall	5.25 ± 1.99 (40)	2.86 ± 1.35 (90)^∗^	1.89 ± 0.92 (83)^∗^^#^	<0.001
Sex				
Male	4.58 ± 1.87 (18)	2.76 ± 1.14 (44)^∗^	1.54 ± 0.74 (38)^∗^^#^	<0.001
Female	5.80 ± 1.96 (22)	2.95 ± 1.54 (46)^∗^	2.18 ± 0.97 (45)^∗^^#†^	<0.001
*P* value	0.052	0.508	0.001	
BMI				
Normal BMI	5.54 ± 1.82 (31)	3.27 ± 1.61 (41)^∗^	2.00 ± 1.03 (35)^∗^^#^	<0.001
Overweight or obese	4.25 ± 2.33 (9)^‡^	2.51 ± 0.98 (49)^∗^^‡^	1.81 ± 0.84 (48)^∗^^#‡^	<0.001
*P* value	0.023	0.011	0.042	

^∗^Significantly different from the NC group. ^#^Significantly different from the NM group. ^†^Significantly different from the male group. ^‡^Significantly different from the normal BMI group. BMI: body mass index. Measurement data are presented as mean ± standard deviation or median (with interquartile ranges). The unit of the concentrations of serum adiponectin: mg/L.

**Table 3:** Clinical characteristics among the different subgroups.

Clinical measurement	High-adiponectin subgroup (*n* = 71)	Moderate-adiponectin subgroup (*n* = 71)	Low-adiponectin subgroup (*n* = 71)	*P* value
Age (y)	52.24 ± 12.15	55.03 ± 10.93	53.37 ± 10.07	0.323
Male, *n* (%)	29 (40.85)	32 (45.07)	39 (54.93)	0.226
Cigarette smoking, *n* (%)	23 (32.39)	25 (35.21)	32 (45.07)	0.262
Alcohol drinking, *n* (%)	44 (61.97)	47 (66.20)	49 (69.01)	0.673
BMI (kg/m^2^)	22.26 ± 2.86	25.00 ± 2.99^∗^	24.74 ± 2.89^∗^	<0.001
Diabetes duration (y)	0.5 (0, 6)^#^	7 (2, 11.5)^∗^	6 (3, 10)^∗^	<0.001
SBP (mmHg)	125 (120, 131)	135 (130, 155)^∗^	155 (132, 160)^∗^	<0.001
DBP (mmHg)	79.90 ± 8.81	84.58 ± 9.19^∗^	87.03 ± 7.89^∗^	<0.001
FPG (mmol/L)	6.10 (5.39, 9.11)	9.08 (7.25, 12.62)^∗^	8.81 (7.92, 10.56)^∗^	<0.001
PPG (mmol/L)	9.86 (6.30, 14.25)	14.60 (11.58, 18.40)^∗^	14.25 (12.30, 17.20)^∗^	<0.001
HbA_1_C (%)	7.33 ± 2.10	8.68 ± 2.00^∗^	8.42 ± 1.57^∗^	<0.001
TC (mmol/L)	4.65 ± 0.93	4.95 ± 1.15	5.11 ± 0.94^∗^	0.024
TG (mmol/L)	1.10 (0.74, 1.68)	1.65 (1.25, 2.17)^∗^	1.48 (1.02, 1.98)^∗^	0.036
HDL-C (mmol/L)	1.29 ± 0.33	1.11 ± 0.32^∗^	1.07 ± 0.25^∗^	<0.001
LDL-C (mmol/L)	3.10 ± 0.82	3.04 ± 0.89	3.52 ± 0.96^∗^^#^	0.003
UA (mmol/L)	316.94 ± 87.70	363.83 ± 80.26^∗^	381.92 ± 84.85^∗^	<0.001
Fg (g/L)	3.45 ± 0.94	3.79 ± 1.08	3.66 ± 0.97	0.133
Fins (*μ*U/mL)	61.65 ± 34.15	103.69 ± 51.78^∗^	128.59 ± 55.18^∗^^#^	<0.001
HOMA2-IR	1.10 (0.60, 1.60)	1.80 (1.50, 3.10)^∗^	2.70 (1.70, 3.50)^∗^	<0.001
hs-CRP (mg/L)	1.01 (0.79, 1.92)	3.04 (0.94, 3.62)^∗^	4.72 (4.20, 6.95)^∗^	<0.001
D-IMT (cm)	0.0451 ± 0.0245	0.0768 ± 0.0337^∗^	0.0946 ± 0.0359^∗^^#^	<0.001
Adiponectin (mg/L)	5.06 ± 1.41	2.46 ± 0.39^∗^	1.27 ± 0.33^∗^^#^	<0.001
Macroangiopathy, *n* (%)	7 (9.86%)	30 (42.25%)	46 (64.79%)	<0.001

^∗^Significantly different from the high-adiponectin level subgroup. ^#^Significantly different from the moderate-adiponectin level subgroup. BMI: body mass index; SBP: systolic blood pressure; DBP: diastolic blood pressure; FPG: fasting plasma glucose; PPG: postprandial 2 hours blood glucose; HbA_1_C: glycosylated hemoglobin; TC: total cholesterol; TG: triglyceride; HDL-C: high-density lipoprotein cholesterol; LDL-C: low-density lipoprotein cholesterol; UA: uric acid; Fg: fibrinogen; Fins: fasting insulin; HOMA2-IR: homeostasis model assessment 2 of insulin resistance; hs-CRP: high-sensitivity C-reactive protein; D-IMT: intima media thickness of the dorsalis pedis artery. Measurement data are presented as mean ± standard deviation or median (with interquartile ranges).

**Table 4:** Partial correlation analysis between adiponectin and relevant clinical indexes.

Variables	Adiponectin		Variables	Adiponectin	
	*r*	*P* value		*r*	*P* value
BMI	-0.391	<0.001	TG	-0.203	0.003
Diabetes duration	-0.394	<0.001	HDL-C	0.305	<0.001
SBP	-0.547	<0.001	LDL-C	-0.170	0.014
DBP	-0.415	<0.001	UA	-0.327	<0.001
FPG	-0.422	<0.001	Fg	-0.193	0.005
PPG	-0.504	<0.001	Fins	-0.540	<0.001
HbA_1_C	-0.423	<0.001	HOMA2-IR	-0.568	<0.001
TC	-0.230	0.001	hs-CRP	-0.372	<0.001
D-IMT	-0.602	<0.001			

*r*: correlation; BMI: body mass index; SBP: systolic blood pressure; DBP: diastolic blood pressure; FPG: fasting plasma glucose; PPG: postprandial 2 hours blood glucose; HbA_1_C: glycosylated hemoglobin; TC: total cholesterol; TG: triglyceride; HDL-C: high-density lipoprotein cholesterol; LDL-C: low-density lipoprotein cholesterol; UA: uric acid; Fg: fibrinogen; Fins: fasting insulin; HOMA2-IR: homeostasis model assessment 2 of insulin resistance; hs-CRP: high-sensitivity C-reactive protein; D-IMT: intima media thickness of the dorsalis pedis artery.

**Table 5:** The odds ratio of risk factors for diabetic macroangiopathy participants versus nondiabetic macroangiopathy participants.

	Model 1	*P* value	Model 2	*P* value	Model 3	*P* value
Age	1.022 (0.955, 1.095)	0.526			1.026 (0.954, 1.104)	0.492
Female	0.215 (0.040, 1.146)	0.072			0.096^∗^ (0.013, 0.690)	0.020
BMI	0.935 (0.728, 1.201)	0.598			0.866 (0.656, 1.144)	0.311
Cigarette smoking	0.627 (0.126, 3.117)	0.569			0.698 (0.129, 3.770)	0.676
HbA_1_C	0.997 (0.659, 1.508)	0.989			1.108 (0.697, 1.763)	0.664
SBP	1.411^∗^ (1.237, 1.609)	<0.00001			1.517^∗^ (1.274, 1.806)	<0.00001
TG	0.985 (0.489, 1.984)	0.966			0.885 (0.425, 1.842)	0.743
HDL-C	0.889 (0.064, 12.267)	0.930			3.095 (0.164, 58.558)	0.451
LDL-C	0.776 (0.365, 1.650)	0.510			0.513 (0.203, 1.296)	0.158
Adiponectin			0.474^∗^ (0.346, 0.648)	<0.00001	0.293^∗^ (0.113, 0.759)	0.011

The odds ratio (OR) and 95% confidence interval of each parameter versus nondiabetic macroangiopathy participants were analyzed using binary logistic regression. Model 1 included traditional macrovascular risk factors such as age, sex, cigarette smoking, body mass index (BMI), HbA_1_C, systolic blood pressure (SBP), triglycerides (TG), high-density lipoprotein cholesterol (HDL-C), and low-density lipoprotein cholesterol (LDL-C) (*n* = 173, ^∗^*P* < 0.05). Model 2 included only adiponectin (*n* = 173, ^∗^*P* < 0.05). Model 3 incorporated Model 1 plus Model 2 (*n* = 173, ^∗^*P* < 0.05).

**Table 6:** Multiple linear regression analysis of relevant risk factors for D-IMT.

	Model 1			Model 2			Model 3		
	*β*	SE	*P*	*β*	SE	*P*	*β*	SE	*P*
Age	-3.45*E* − 5	0.000	0.856				-2.01*E* − 5	0.000	0.912
Female	-0.002	0.004	0.558				-0.001	0.004	0.822
BMI	0.001	0.001	0.405				9.61*E* − 5	0.001	0.871
Cigarette smoking	-0.007	0.004	0.106				-0.008	0.004	0.067
HbA_1_C	-0.001	0.001	0.291				-0.001	0.001	0.307
SBP	0.001	0.000	0.000				0.001	0.000	0.000
TG	-0.003	0.002	0.198				-0.003	0.002	0.206
HDL-C	-0.007	0.007	0.291				-0.003	0.007	0.613
LDL-C	0.001	0.002	0.758				8.63*E* − 5	0.002	0.966
Adiponectin				-0.011	0.002	0.000	-0.006	0.002	0.000

D-IMT: intima media thickness of the dorsalis pedis artery; *β*: unstandardized coefficients; SE: standard error. Model 1 included traditional macrovascular risk factors such as age, sex, cigarette smoking, body mass index (BMI), HbA_1_C, systolic blood pressure (SBP), triglycerides (TG), high-density lipoprotein cholesterol (HDL-C), and low-density lipoprotein cholesterol (LDL-C) (*n* = 173,^∗^*P* < 0.05). Model 2 included only adiponectin (*n* = 173, ^∗^*P* < 0.05). Model 3 incorporated Model 1 plus Model 2 (*n* = 173, ^∗^*P* < 0.05).

## Data Availability

The data used to support the findings of this study are available from the corresponding author upon request.

## References

[1] Haffner S. M., Lehto S., Rönnemaa T., Pyörälä K., Laakso M. (1998). Mortality from coronary heart disease in subjects with type 2 diabetes and in nondiabetic subjects with and without prior myocardial infarction. *The New England Journal Medicine*.

[2] Ko G. T., Chow C. C., Leung G. (2011). High rate of increased carotid intima-media thickness and atherosclerotic plaques in Chinese asymptomatic subjects with central obesity. *International Journal of Cardiovascular Imaging*.

[3] Voulgari C., Papadogiannis D., Tentolouris N. (2010). Diabetic cardiomyopathy: from the pathophysiology of the cardiac myocytes to current diagnosis and management strategies. *Vascular Health and Risk Management*.

[4] Salonen J. T., Salonen R. (1993). Ultrasound B-mode imaging in observational studies of atherosclerotic progression. *Circulation*.

[5] Miyamoto M., Kotani K., Okada K. (2012). The correlation of common carotid arterial diameter with atherosclerosis and diabetic retinopathy in patients with type 2 diabetes mellitus. *Acta Diabetologica*.

[6] Saba L., Ikeda N., Deidda M. (2013). Association of automated carotid IMT measurement and HbA1c in Japanese patients with coronary artery disease. *Diabetes Research and Clinical Practice*.

[7] Qi L. X., Gu Y. Q., Yu H. X., Li X. F., Cui S. J., Guo L. R. (2005). Comparison of angiographic characteristics in lower extremity between nondiabetic and diabetic atheroscleroses with analysis of its clinical significance. *Chinese Journal of Diabetes*.

[8] Kizu A., Koyama H., Tanaka S. (2003). Arterial wall stiffness is associated with peripheral circulation in patients with type 2 diabetes. *Atherosclerosis*.

[9] Premanath M., Raghunath M. (2010). Ankle-brachial index by oscillometry: a very useful method to assess peripheral arterial disease in diabetes. *International Journal of Diabetes in Developing Countries*.

[10] Zhao Y. W., Gan X. Y., Li J. S. (2002). Evaluation of diagnosing pathological changes in lower limb small and medium arteries of diabetic patients by color Doppler ultrasound. *Oncoradiology*.

[11] Wang A. H., Xu Z. R., Wang Y. Z. (2005). Diabetic patients with unpalpable dorsalis pedis artery pulse have more macrovascular risk factors. *Negative*.

[12] Kishida K., Funahashi T., Shimomura I. (2014). Adiponectin as a routine clinical biomarker. *Best Practice & Research Clinical Endocrinology & Metabolism*.

[13] Arita Y., Kihara S., Ouchi N. (2012). Paradoxical decrease of an adipose-specific protein, adiponectin, in obesity. *Biochemical and Biophysical Research Communications*.

[14] Cinar N., Gurlek A. (2013). Association between novel adipocytokines adiponectin, vaspin, visfatin, and thyroid: an experimental and clinical update. *Endocrine Connections*.

[15] Mangge H., Almer G., Truschnig-Wilders M., Schmidt A., Gasser R., Fuchs D. (2010). Inflammation, adiponectin, obesity and cardiovascular risk. *Current Medicinal Chemistry*.

[16] Department of Noncommunicable Disease Surveillance (1999). *Definition, diagnosis and classification of diabetes mellitus and its complications: report of a WHO consultation. Part 1: diagnosis and classification of diabetes mellitus*.

[17] Wallace T. M., Levy J. C., Matthews D. R. (2004). Use and abuse of HOMA modeling. *Diabetes Care*.

[18] Wildman R. P., Gu D., Reynolds K., Duan X., He J. (2004). Appropriate body mass index and waist circumference cutoffs for categorization of overweight and central adiposity among Chinese adults. *American Journal of Clinical Nutrition*.

[19] Yang W., Lu J., Weng J. (2010). Prevalence of diabetes among men and women in China. *The New England Journal of Medicine*.

[20] Paolisso G., Howard B. V. (1998). Role of non-esterified fatty acids in the pathogenesis of type 2 diabetes mellitus. *Diabetic Medicine*.

[21] Jude E. B., Oyibo S. O., Chalmers N., Boulton A. J. (2001). Peripheral arterial disease in diabetic and nondiabetic patients: a comparison of severity and outcome. *Diabetes Care*.

[22] Braicu M. D., Priţulescu C., Alexandru D., Moţa M. (2009). The assessment of subclinic atherosclerosis objected through IMT in normal and dyslipidemic patients with various degrees of glucose tolerance. *Romanian Journal of Internal Medicine*.

[23] Gan L., Ye Z., Chen L., Liu X. X. (2008). Relationship between the arteriosclerosis of dorsalis pedis and plasma adiponectin in type 2 diabetes. *Journal of Fujian Medical University*.

[24] Yamauchi T., Kamon J., Ito Y. (2003). Cloning of adiponectin receptors that mediate anti-diabetic metabolic effects. *Nature*.

[25] Shetty S., Kusminski C. M., Scherer P. E. (2009). Adiponectin in health and disease: evaluation of adiponectin-targeted drug development strategies. *Trends in Pharmacological Sciences*.

[26] Kadowaki T., Yamauchi T. (2011). Adiponectin receptor signaling: a new layer to the current model. *Cell Metabolism*.

[27] Turer A. T., Browning J. D., Ayers C. R. (2012). Adiponectin as an independent predictor of the presence and degree of hepatic steatosis in the Dallas Heart Study. *Journal of Clinical Endocrinology Metabolism*.

[28] Li Y., Wu Q. H., Jiao M. L. (2015). Gene-environment interaction between adiponectin gene polymorphisms and environmental factors on the risk of diabetic retinopathy. *Journal of Diabetes Investigation*.

[29] Matsuda M., Shimomura I., Sata M. (2002). Role of adiponectin in preventing vascular stenosis. The missing link of adipo-vascular axis. *Journal of Biological Chemistry*.

[30] Okamoto Y., Kihara S., Ouchi N. (2002). Adiponectin Reduces Atherosclerosis in Apolipoprotein E-Deficient Mice. *Circulation*.

[31] Ekmekci H., Ekmekci O. B. (2006). The role of adiponectin in atherosclerosis and thrombosis. *Clinical and Applied Thrombosis Hemostasis*.

[32] Hui E., Xu A., Chow W. S. (2014). Hypoadiponectinemia as an independent predictor for the progression of carotid atherosclerosis: a 5-year prospective study. *Metabolic Syndrome and Related Disorders*.

[33] Zampetaki A., Kirton J. P., Xu Q. (2008). Vascular repair by endothelial progenitor cells. *Cardiovascular Research*.

[34] Sambuceti G., Morbelli S., Vanella L. (2009). Diabetes impairs the vascular recruitment of normal stem cells by oxidant damage, reversed by increases in pAMPK, heme oxygenase-1, and adiponectin. *Stem Cells*.

[35] Libby P., Okamoto Y., Rocha V. Z., Folco E. (2010). Inflammation in atherosclerosis: transition from theory to practice. *Circulation Journal*.

[36] Cheng X., Folco E. J., Shimizu K., Libby P. (2012). Adiponectin induces pro-inflammatory programs in human macrophages and CD4+ T cells. *Journal of Biological Chemistry*.

[37] Weyer C., Funahashi T., Tanaka S. (2001). Hypoadiponectinemia in obesity and type 2 diabetes: close association with insulin resistance and hyperinsulinemia. *Journal of Clinical Endocrinology and Metabolism*.

[38] Tishinsky J. M., Dyck D. J., Robinson L. E. (2012). Lifestyle factors increasing adiponectin synthesis and secretion. *Vitamins and Hormones*.

[39] Nishizawa H., Shimomura I., Kishida K. (2002). Androgens decrease plasma adiponectin, an insulin-sensitizing adipocyte-derived protein. *Diabetes*.

[40] Cicero A. F. G., Magni P., Moré M. (2011). Adipokines and sexual hormones associated with the components of the metabolic syndrome in pharmacologically untreated subjects: data from the Brisighella Heart Study. *International Journal of Endocrinology*.

[41] Dougherty A. H. (2011). Gender balance in cardiovascular research: importance to women’s health. *Texas Heart Institute Journal*.

[42] Chakrabarti S., Morton J. S., Davidge S. T. (2014). Mechanisms of estrogen effects on the endothelium: an overview. *Canadian Journal of Cardiology*.

[43] Fariba F., Moradi M., Arabi A., Ghaderi B. (2016). Prevalence of coronary artery ectasia with atherosclerosis and associated risk factors in the west of Iran: a cross-sectional study. *Journal of Research in Health Sciences*.

[44] Patel A., ADVANCE Collaborative Group (2007). Effects of a fixed combination of perindopril and indapamide on macrovascular and microvascular outcomes in patients with type 2 diabetes mellitus (the ADVANCE trial): a randomised controlled trial. *The Lancet*.

[45] The ACCORD Study Group (2010). Effects of intensive blood-pressure control in type 2 diabetes mellitus. *The New England Journal of Medicine*.

[46] Lihn A. S., Østergård T., Nyholm B., Pedersen S. B., Richelsen B., Schmitz O. (2003). Adiponectin expression in adipose tissue is reduced in first-degree relatives of type 2 diabetic patients. *American Journal of Physiology-Endocrinology and Metabolism*.

[47] Kowalska I., Straczkowski M., Nikołajuk A. (2006). Plasma adiponectin and E-selectin concentrations in patients with coronary heart disease and newly diagnosed disturbances of glucose metabolism. *Advances in Medical Sciences*.

[48] Kablak-Ziembicka A., Przewlocki T., Sokołowski A., Tracz W., Podolec P. (2011). Carotid intima-media thickness, hs-CRP and TNF-*α* are independently associated with cardiovascular event risk in patients with atherosclerotic occlusive disease. *Atherosclerosis*.

